# Liquid phase condensation directs nucleosome epigenetic modifications

**DOI:** 10.1038/s41392-020-0166-2

**Published:** 2020-05-06

**Authors:** Yi Yang, Chuanhe Yu

**Affiliations:** The Hormel Institute, University of Minnesota, Austin, MN 55912 USA

**Keywords:** Epigenetics, Epigenetics

In a recent study published in *Nature*, Gallego et al. provided an interesting model in which layered condensates of histone-modifying enzymes generate chromatin-associated “reaction chambers” to help performing catalytic activity along gene bodies.^1^

Eukaryotic transcription is regulated by chromatin structure, whose alterations are mediated by conserved post-translational histone tail modifications. The consequences of these epigenetic modifications have been linked to cell development, cell cycle regulation, as well as cell state and fate. One of these modifications is ubiquitylation (ub), which adds a polypeptide (ubiquitin) to a lysine residue of histone. Ubiquitylation reaction requires a ubiquitin-activating enzyme (E1), a ubiquitin-conjugating enzyme (E2), and a ubiquitin-ligating enzyme (E3). H2BK120ub is ubiquitylated H2B at position 120 in human, and has been found to be important for gene transcriptional regulation in cell differentiation, organism development, and tumorigenesis.^2^

The orthologous residue for ubiquitylated H2B in yeast is H2BK123. H2BK123ub is generated by Bre1 (E3), with Rad6 (E2), and Uba1 (E1). Generally, the level of H2BK123ub correlates with the elongation rate of RNA polymerase II. The protein Large 1 (Lge1), which regulates the size of yeast cell, can copurify with Bre1 and is essential for H2B mono-ubiquitylation. However, its mechanistic role in this modification is unknown.^3^ In order to explore the function of Lge1 on H2BK123ub, Gallego et al. analyzed the protein sequence of Lge1 and identified an intrinsically disordered region (IDR). This region likely participates in multivalent interactions and may lead to liquid–liquid phase separation (LLPS) and the formation of biomolecular condensates. In this study, the in vitro LLPS assay shows that Lge1 protein itself could form condensates. When Bre1 was mixed with Lge1, Bre1 formed a distinct compartment encapsulating the Lge1 core. Bre1 and Lge1 directly interacted with each other and formed large complexes in cells. To address the function of LLPS on the H2B ubiquitylation in vivo, Gallego et al. performed H2BK123ub ChIP-exo analysis to provide a high-resolution location pattern within each nucleosome. The results suggest that the IDR of Lge1 enhances H2BK123ub at the gene body region but not at the transcriptional +1 nucleosome region. While H2BK123ub at the transcriptional +1 position does not depend on the Lge1 liquid–liquid phase separation, an unknown mechanism is likely involved in the ubiquitylation regulation at this position. Interestingly, Gallego et al. found that substituting the IDR region of yeast Lge1 with a similar IDR region from WAC, a human Lge1 counterpart, was functional on H2Bub. It suggests the evolutionary conservation of Lge1-mediated LLPS on directing regulation of transcriptional H2BK123ub. As the histone variant H2A.Z is assembled into the transcriptional +1 position, the authors further tested the genetic interaction between H2A.Z and the IDR region of Lge1. The results indicate that LLPS of Lge1 is essential for viability when H2A.Z encoding gene Htz1 is absent from +1 nucleosomes, and also reinforce the physiological importance of Lge1-Bre1 condensates. Overall, this study demonstrates that the Bre1 shell, facilitated by Lge1-mediated LLPS, has a direct catalytic role on nucleosome ubiquitylation. Given that mutations in human Lge1 orthologue WAC could lead to DeSanto–Shinawi syndrome (a neurodevelopmental disorder), this study also implies that LLPS might contribute to this syndrome.

**Fig. 1:**
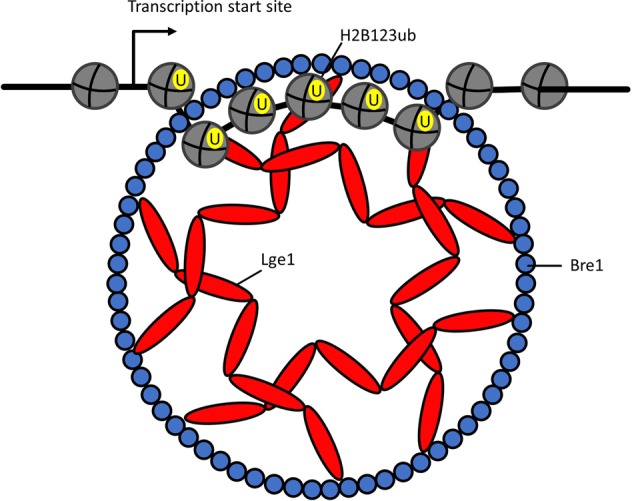
Lipid phase separation mediated by the scaffold protein Lge1 guides H2B ubiquitination modification. Lge1 protein has an intrinsically disordered region (IDR) and can lead to the formation of biomolecular condensates. E3 ligase Bre1, which encapsulates the Lge1 core, can ubiquitinylate H2BK123 of a nucleosome with other ubiquitylation enzymes in a co-transcriptional way

An important question regarding the chromatin-associated “reaction chambers” model is how the protein factors enter and leave the chamber in a controlled manner. In this study, the E3 ligase Bre1 was found locating at the shell of Lge1-mediated chamber (Fig. 1). The E2 ligase Brad6 can only slowly enter the reaction chamber, while the nucleosome-associated chromatin seems to enter the chamber freely. This study shows that H2BK123ub colocalizes with the transcriptional regions. Likely the transcriptional machinery plays a role in chromatin association with Lge1-mediated chamber. Whether the composition or the physiological nature of the large biomolecular plays a fundamental role in controlling the “in and out” is still unknown. More studies in this direction will clarify the process behind.

LLPS condensates have been shown to be involved in multiple cellular processes and functions. Over the past several years, researchers in the chromatin field have begun to draw attention to LLPS. Several chromatin proteins, including heterochromatin protein 1 (HP1), bromodomain-containing protein 4 (Brd4) and polycomb repressive complex 1 (PRC1) components have an intrinsic capacity for LLPS.^4–8^ Through LLPS, nuclear chromatin can be compartmentalized. All these studies begin to shed light on the relationship between intercellular spatial compartmentalization and chromatin structure. The significance of this study is the original discovery that the intercellular LLPS is involved in the regulation of epigenetic enzymatic activity at specific genomic regions and has a substantial impact on biological and cellular functions. It is hard to imagine how widely LLPS affects the epigenetic regulation program. As the intrinsically disordered proteins (IDPs) and intrinsically disordered proteins regions (IDPRs) are highly abundant throughout different organisms, more proteins with this structure may mediate the epigenetic enzymatic ‘reaction chambers’ structure and play a role in the epigenetic regulation process.^9^ Another key point about the intercellular LLPS on the epigenetic regulation is that it provides separated local environments for complex epigenetic reactions in addition to the cellular organelle compartments, like endoplasmic reticulum, Golgi apparatus, nucleus, and mitochondria etc. Many important cellular functions are performed in a stepwise pathway. One good example is the new histone H3-H4 tetramer assembly pathway, in which newly synthesized histone H3-H4 sequentially undergoes acetylation (H3K56Ac) and ubiquitylation (H3K121,123,125ub) which will promote the deposition of H3-H4 tetramer onto chromatin.^10^ Although the biochemical experiments strongly support H3-H4 tetramer histone acetylation and ubiquitylation crosstalk model, how the stepwise processes are separated and controlled is unknown. The intercellular LLPS provides a reasonable explanation—two different enzymatical reaction chambers undergo acetylation and ubiquitylation reactions. In such a situation, histone tetramer will be transferred between two isolated enzymatic chambers, possibly by histone chaperons. In this view, the liquid phase condensation model provides a lot of insights into the orchestration of complex epigenetic enzyme reactions and offers good opportunities for future exploration.
